# Semaglutide for type 2 diabetes

**DOI:** 10.18773/austprescr.2020.046

**Published:** 2020-07-16

Approved indication: type 2 diabetes

Ozempic (Novo Nordisk)

pre-filled pens containing 1.34 mg/mL solution

When type 2 diabetes is no longer controlled by diet, exercise and metformin there are many options for additional treatment. These options include the glucagon-like peptide-1 (GLP-1) analogues such as dulaglutide and exenatide. When there is hyperglycaemia, these agonists act on GLP-1 receptors in the pancreas to increase insulin secretion.^1^

Semaglutide is another genetically engineered GLP-1 receptor agonist. As a peptide, it has to be given by subcutaneous injection. The half-life of semaglutide is approximately one week, so it only needs to be injected once a week. A steady state is reached after 4–5 weeks of weekly injections. It is cleared like other peptides, so excretion should not be affected by renal or hepatic impairment.

Semaglutide has been studied in a series of trials titled the Semaglutide Unabated Sustainability in Treatment of type 2 diabetes (SUSTAIN). These phase III trials assessed the effect of weekly injections on concentrations of glycated haemoglobin (HbA1c) (see Table).^2^-^9^

**Table:** Efficacy of semaglutide weekly injections in type 2 diabetes

Trial (design)	Duration (weeks)	Baseline drug therapy	Patient allocations			Mean baseline HbA1c mmol/mol (%)	Mean decrease in HbAlc mmol/mol (%) at end of trial
SUSTAIN 1^2^ (double-blind)	30	None	Semaglutide	0.5 mg	129	64.88 (8.09%)	15.9 (1.45%)
				1 mg	130	65.29 (8.88%)	16.96 (1.55%)
			Placebo		129	63.43 (7.95%)	0.27 (0.02%)
SUSTAIN 2^3^ (double-blind)	56	Metformin with or without thiazolidinediones	Semaglutide	0.5 mg	409	64.1 (8.0%)	14.4 (1.3%)
				1 mg	409	64.4 (8.0%)	17.6 (1.6%)
			Sitagliptan	100 mg	407	65.8 (8.2%)	6.0 (0.5%)
SUSTAIN 3^4^ (open-label)	56	One or two of: metformin, sulfonylureas, thiazolidinediones	Semaglutide	1 mg	404	67.9 (8.4%)	16.8 (1.5%)
			Exenatide	2 mg	405	67.6 (8.3%)	10.0 (0.9%)
SUSTAIN 4^5^ (open-label)	30	Metformin with or without sulfonylureas	Semaglutide	0.5 mg	362	65.4 (8.1%)	13.22 (1.21%)
				1 mg	360	66.6 (8.3%)	17.93 (1.64%)
			Insulin glargine	360	360	65.4 (8.1%)	9.06 (0.83%)
SUSTAIN 5^6^ (double-blind)	30	Insulin (basal) with or without metformin	Semaglutide	0.5 mg	132	67.9 (8.4%)	15.8 (1.4%)
				1 mg	131	67.3 (8.3%)	20.2 (1.8%)
			Placebo		133	68.6 (8.4%)	1.0 (0.1%)
SUSTAIN 6^7^ (double-blind)	104	Up to two oral drugs with or without insulin	Semaglutide	0.5 mg	826	72 (8.7%)	12.1 (1.1%)
				1 mg	822	72 (8.7%)	15.8 (1.4%)
			Placebo		1649	72 (8.7%)	5.0 (0.4%)
SUSTAIN 7^8^ (open-label)	40	Metformin	Semaglutide	0.5 mg	301	67.5 (8.3%)	16.5 (1.5%)
				1 mg	300	66.2 (8.2%)	19.4 (1.8%)
			Dulaglutide	0.75 mg	299	65.7 (8.2%)	12.1 (1.1%)
					299	66.1 (8.2%)	14.9 (1.4%)
SUSTAIN 8^9^ (double-blind)	52	Metformin	Semaglutide	1 mg	394	67.1 (8.3%)	16.0 (1.5%)
			Canagliflozin	300 mg	394	66.3 (8.2%)	10.7 (1%)

HbA1c glycated haemoglobin

## Monotherapy

Although semaglutide will probably be a second-line treatment, it has been approved as monotherapy when metformin is contraindicated or cannot be tolerated. In the SUSTAIN 1 trial, 388 previously untreated patients were randomised to receive semaglutide (0.5 mg or 1 mg) or a placebo. At the start of the study the mean HbA1c was 64.54 mmol/mol (8.05%). In the 259 patients randomised to semaglutide the HbA1c fell by 15.9–16.96 mmol/mol (1.45–1.55%) after 30 weeks. There was minimal change in the placebo group. Patients treated with semaglutide lost 3.7–4.5 kg in weight.^2^

## Added to metformin monotherapy

SUSTAIN 8 studied 788 patients with diabetes that was not controlled by metformin. They were randomised to receive either semaglutide 1 mg or canagliflozin 300 mg. The average baseline HbA1c concentration was 66.7 mmol/mol (8.3%). After one year this had declined by 16 mmol/mol (1.5%) with semaglutide and 10.7 mmol/mol (1%) with canagliflozin. There was a weight loss of 5.3 kg with semaglutide and 4.2 kg with canagliflozin.^9^

## Added to metformin (with or without sulfonylureas)

SUSTAIN 4 was an open-label trial that compared adding weekly semaglutide (0.5 mg or 1 mg) or once-daily insulin glargine to the treatment of 1082 patients with inadequately controlled diabetes (mean HbA1c 65.8 mmol/mol (8.2%)). Metformin monotherapy was being used by 523 patients while 559 were also taking a sulfonylurea. At week 30 the mean HbA1c concentration had declined by 13.22–17.93 mmol/mol (1.21–1.64%) with semaglutide, while adding insulin reduced it by 9.06 mmol/mol (0.83%). Patients injecting semaglutide lost weight while those injecting insulin gained weight.^5^

## Added to metformin (with or without a thiazolidinedione)

SUSTAIN 2 enrolled 1231 patients who had insufficient glycaemic control despite treatment with metformin, a thiazolidinedione or both. They were randomised to add semaglutide (0.5 mg or 1 mg) or daily sitagliptin (100 mg), an inhibitor of dipeptidyl peptidase-4. After 56 weeks, from a baseline of 64.8 mmol/mol (8.1%), the HbA1c concentration had fallen by 14.4–17.6 mmol/mol (1.3–1.6%) with semaglutide. The reduction with sitagliptin was 6 mmol/mol (0.5%). Patients injected with semaglutide lost 2.4–4.2 kg more weight than the sitagliptin group.^3^

## Added to insulin

SUSTAIN 5 studied the effect of adding semaglutide to the treatment of 397 patients with diabetes that was being managed with basal insulin. Most of these patients (83%) were also taking metformin, but still had an average HbA1c concentration of 67.9 mmol/mol (8.4%). The patients were randomised to take semaglutide (0.5 mg or 1 mg) or a placebo for 30 weeks. At the end of the trial HbA1c had been reduced by 15.8–20.2 mmol/mol (1.4–1.8%) with semaglutide compared with a reduction of 1 mmol/mol (0.1%) in the placebo group. Compared to the reduction in weight in the placebo group (1.4 kg), patients injecting semaglutide lost an extra 2.3–5.1 kg.^6^

## Comparison with other GLP-1 agonists

SUSTAIN 3 compared semaglutide 1 mg to weekly injections of exenatide 2 mg. The 813 patients in this open-label trial had an average HbA1c of 67.7 mmol/mol (8.3%) despite taking one or two oral antidiabetic drugs. After 56 weeks this concentration had declined by 16.8 mmol/mol (1.5%) with semaglutide and by 10 mmol/mol (0.9%) with exenatide. Body weight reduced by 5.6 kg with semaglutide and by 1.9 kg with exenatide.^4^

Semaglutide (0.5 mg or 1 mg) was compared to weekly injections of dulaglutide (0.75 mg or 1.5 mg) in the open-label SUSTAIN 7 trial. This randomised 1201 patients who had an average HbA1c concentration of approximately 66.4 mmol/mol (8.2%) despite taking metformin. After 40 weeks the reductions in HbA1c were 16.5 mmol/mol (1.5%) with semaglutide 0.5 mg and 12.1 mmol/mol (1.1%) with dulaglutide 0.75 mg. The corresponding reductions for semaglutide 1 mg and dulaglutide 1.5 mg were 19.4 mmol/mol (1.8%) and 14.9 mmol/mol (1.4%). Depending on the dose, weight loss with semaglutide was 4.6–6.5 kg and 2.3–3 kg with dulaglutide.^8^

## Safety

Some of the adverse effects of semaglutide can be predicted from its mechanism of action.^1^ For example, there is a risk of hypoglycaemia when semaglutide is given with insulin or a sulfonylurea. Treatment with semaglutide should begin at a low dose and be increased after four weeks. As GLP-1 receptors are found in the brain, heart and kidneys, as well as in the pancreas, semaglutide may have effects on these organs. For example, semaglutide has been associated with an increase in heart rate. It delays gastric emptying. Gastrointestinal events such as nausea, vomiting and diarrhoea are the most frequent adverse reactions. Withdrawals due to adverse events varied across trials. In SUSTAIN 1 approximately 6% of patients taking semaglutide withdrew because of adverse events compared with approximately 2% of the placebo group.^2^ Less frequent adverse effects include acute pancreatitis, cholethiasis and complications of diabetic retinopathy. Injecting a peptide can cause an immune response. In addition to injection site reactions, anaphylaxis has been reported rarely. Laboratory tests show that semaglutide may increase concentrations of lipase and amylase.

In animal studies semaglutide has been toxic during pregnancy. It should not be used by pregnant or breastfeeding women.

## Place in therapy

Semaglutide is an option when the use of a GLP-1 analogue is considered. This will usually be if drug therapy with metformin is insufficient to control type 2 diabetes. In the open-label trials the absolute differences between semaglutide and exenatide^4^ and dulaglutide^8^ were small, but they met the criteria for statistical superiority for the reductions in HbA1c and body weight. While increasing the dose of semaglutide to 1 mg will cause a slightly greater reduction of HbA1c it will also increase adverse effects.

Changes in the concentrations of HbA1c are a surrogate outcome in type 2 diabetes. It is too early to assess all the long-term clinical outcomes, however semaglutide might have some benefit in patients with a high risk of cardiovascular events. SUSTAIN 6 enrolled 3297 patients with cardiovascular disease, chronic heart failure or chronic kidney disease. These patients had an average HbA1c concentration of 72 mmol/mol (8.7%). They were randomised to semaglutide (0.5 mg or 1 mg) or a placebo. After a median follow-up of 2.1 years there had been a cardiovascular event in 6.6% of the semaglutide group and 8.9% of the placebo group. However, deaths from cardiovascular causes were similar (2.7% vs 2.8%) in both groups. The patients injecting semaglutide also had more complications from diabetic retinopathy (3.0 vs 1.8%).^7^

An oral formulation of semaglutide has been developed. If this is approved for use in Australia, it may give semaglutide an advantage over the other GLP-1 analogues.


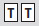
 manufacturer provided additional useful information

The Transparency Score is explained in New drugs: transparency, Aust Prescr 2014;37:27.

At the time the comment was prepared, information about this drug was available on the websites of the Food and Drug Administration in the USA, and the European Medicines Agency.
